# COSTS FOR THE SURGICAL TREATMENT OF OBESITY THROUGH LAPAROSCOPY IN A FEDERAL TERTIARY HOSPITAL BY THE BRAZILIAN UNIFIED HEALTH SYSTEM

**DOI:** 10.1590/0102-6720202400042e1836

**Published:** 2024-12-02

**Authors:** Álvaro Antonio Bandeira FERRAZ, Hiago Dantas MEDEIROS, Fernando SANTA-CRUZ, Flávio KREIMER

**Affiliations:** 1 Universidade Federal de Pernambuco, Department of Surgery - Recife (PE), Brazil; 2 Universidade Federal de Pernambuco, University Hospital, General Surgery Residence - Recife (PE), Brazil; 3 Hospital dos Servidores do Estado, General Surgery Residence - Recife (PE), Brazil

**Keywords:** Bariatric surgery, Obesity, Gastric bypass, Hospital costs., Cirurgia bariátrica, Oesidade, Derivação gástrica, Custos hospitalares.

## Abstract

**BACKGROUND:**

Obesity is a multifactorial disease affecting a significant portion of the population. Bariatric surgery emerges as a prominent approach in this context, representing an effective treatment both in the short and long term. The costs associated with bariatric surgery vary depending on the characteristics of the patients, current hospital practices, and available funding sources.

**AIMS:**

To analyze the costs of minimally invasive bariatric surgery for the treatment of obesity in a tertiary federal public hospital.

**METHODS:**

An observational and descriptive study aimed at assessing the costs associated with laparoscopic vertical gastrectomy (GV) and Roux-en-Y gastric bypass (RYGB) in a federal public tertiary service from 2018 to 2021. Data were obtained through the management of medical-hospital expenses related to surgical and anesthetic supplies, as well as the amount reimbursed by the funding source to the hospital.

**RESULTS:**

Over the analyzed period, a total of 177 minimally invasive bariatric surgeries were performed. In terms of the charges, since 2018, the hospital has been receiving an amount of R$ 6,145.00 for the “bariatric surgery by videolaparoscopy” procedure, which includes RYGB, and R$ 4,095.00 for “vertical gastrectomy.” Regarding the average hospital cost of surgical supplies, RYGB incurred a total of R$ 9,907.54, while GV incurred a total of R$ 9,315.84. The average total cost of RYGB was R$ 10,799.23, and, for GV, it was R$ 10,207.53. These figures indicate that the hospital incurred a loss of approximately R$ 4,654.23 for performing RYGB and R$ 6,112.53 for GV.

**CONCLUSION:**

Despite the increasing number of eligible patients for surgical treatment of obesity and the consequent quantitative growth of these procedures funded by the Brazilian Unified Health System (SUS), the costs exceed the reimbursement from the funding source in federal public hospitals. There is a need for a precise assessment of financing in the fight against obesity.

## RESUMO

**RACIONAL:**

A obesidade é uma doença multifatorial que afeta uma parcela significativa da população. A cirurgia bariátrica surge como uma abordagem proeminente neste contexto, representando um tratamento eficaz tanto a curto quanto a longo prazo. Os custos associados à cirurgia bariátrica variam dependendo das características dos pacientes, das práticas hospitalares atuais e das fontes de financiamento disponíveis.

**OBJETIVOS:**

Analisar os custos da cirurgia bariátrica minimamente invasiva para tratamento de obesidade em hospital terciário federal em doentes do Sistema único de Saúde (SUS).

**MÉTODOS:**

Estudo observacional e descritivo que se propôs a avaliar os custos relacionados à realização da gastrectomia vertical (GV) e do Bypass gástrico em Y de Roux (BGYR) por via laparoscópica, no período de 2018 a 2021. Os dados foram obtidos através da gestão de despesas médico-hospitalares sobre custos relacionados a insumos cirúrgicos e anestésicos, bem como o valor repassado pela fonte provedora ao hospital.

**RESULTADOS:**

Ao longo do período analisado, foram contabilizadas 177 cirurgias bariátricas minimamente invasivas. No que diz respeito aos valores cobrados, desde 2018, o hospital recebe um montante de R$ 6.145,00 pelo procedimento “cirurgia bariátrica por videolaparoscopia”, onde incluía-se o BGYR, e R$ 4095,00 pela “gastrectomia vertical”. Em relação ao custo médio hospitalar com insumos cirúrgicos, o BGYR dispendeu um total de R$ 9.907,54, enquanto a GV dispendeu um total de R$ 9.315,84. O valor total médio do BGYR foi de R$ 10.799,23 e da GV foi de R$ 10.207,53. Esses valores indicam que o hospital teve um prejuízo de cerca de R$ 4.654,23 para realizar BGYR e R$ 6.112,53 para a GV.

**CONCLUSÕES:**

Apesar do crescente número de pacientes elegíveis para o tratamento cirúrgico da obesidade e, consequente aumento quantitativo desses procedimentos financiados pelo SUS, os gastos ultrapassam o repasse da fonte provedora, havendo necessidade de uma avaliação precisa do financiamento em prol do combate a obesidade.

## INTRODUCTION

**Central Message**

Bariatric surgery has proven to be effective in promoting sustained weight loss in obese patients, controlling associated comorbidities, and reducing mortality. The laparoscopic approach was included in the Brazilian Unified Health System (SUS) procedure list in 2017. In this context, combined with the predominant dependence of the Brazilian population on SUS, a comprehensive evaluation of the costs inherent to this procedure is important, with the goal of providing cost-effective care to all those with a formal indication for bariatric surgery.

**Perspectives**

The growing number of patients eligible for surgical treatment of obesity, and the consequent increase in the number of procedures funded by the SUS, has led to hospital costs exceeding the reimbursement from the funding source in federal public hospitals, resulting in a negative financial balance.

Obesity is a multifactorial disease with a chronic and progressive course, affecting a significant portion of the population and reaching pandemic status since the early 21^st^ century^1,7^. This global health problem has considerable socioeconomic implications for healthcare systems, given the current trend of increasing public expenditures related to patients affected by obesity. This rise is correlated with the greater use of healthcare services and additional spending on medications, particularly for the treatment of associated comorbidities^8,13^.

The negative impact of obesity on labor productivity, combined with absenteeism in the workplace, significantly contributes to the socioeconomic burden of this health condition. This, along with the associated morbidity and mortality, particularly related to cardiovascular complications, justifies the substantial financial strain placed on healthcare providers, whether public or private^9^. It is estimated that the global economic impact of obesity is around $2.0 trillion, representing approximately 2.8% of the world’s Gross Domestic Product (GDP)^17^.

Given the growing demand for healthcare services, the resources available for such purposes are subject to increasing constraints, making it imperative to promote their rational use^2^. Bariatric surgery emerges as a prominent approach in this context, representing an effective treatment both in the short and long term for severe obesity^14^. This intervention has proven effective in promoting sustained weight loss, controlling associated comorbidities, and reducing mortality in this population. Recent research suggests the early indication of the surgical procedure, despite its inherent risks, as a key strategy, especially in managing endocrinopathies and renal diseases^9^. The costs associated with bariatric surgery vary depending on the characteristics of the patient population, current hospital practices, and available funding sources^15^. Interestingly, evidence points to higher expenses within the public health system compared to the private sector^11^.

In the Brazilian context, laparoscopic bariatric surgery was included in the Unified Health System (SUS) procedure list through Ordinance No. 482, dated March 6, 2017, issued by the Ministry of Health. Since then, SUS has reimbursed accredited hospitals with R$ 3,259.72 per procedure. In 2017, this amount was adjusted to R$ 6,145.00. However, this reimbursement still falls short of the actual hospital costs^10^. Brazil currently ranks second globally in the number of bariatric surgeries performed, according to data from the International Federation for the Surgery of Obesity and Metabolic Disorders (IFSO)^9^. Given the predominant dependence of the Brazilian population on the public health system, there is an urgent need for a comprehensive evaluation of the costs inherent to this procedure to provide cost-effective care for all those with a formal indication for bariatric surgery within the public health system. The scope of this study is to conduct a descriptive analysis of the costs related to performing laparoscopic bariatric surgery at a federal tertiary hospital, comparing them with the actual reimbursement amounts per procedure.

## METHODS

An observational descriptive study conducted at the University Hospital of the Universidade Federal de Pernambuco (HC/UFPE-EBSERH) aimed to examine the costs associated with laparoscopic surgical treatment of obesity, including the two most commonly used techniques - Roux-en-Y gastric bypass (RYGB) and sleeve gastrectomy (SG). The study sought to identify the factors with the greatest financial impact on the final cost of the procedures analyzed and determine the funding contribution of the service relative to the amount reimbursed by the funding source.

Data from all bariatric surgeries performed at this center from 2018 to 2021 were collected using the institution’s own database. Revisional surgeries or those for treating postoperative complications of patients initially subjected to any of the evaluated surgical modalities were excluded. The study protocol was approved by the Institution’s Ethics Committee for Research Involving Human Beings (Certificate of Presentation for Ethical Appreciation - CAAE 73812523.7.0000.5208).

The sample was selected non-probabilistically, for convenience, and consisted of 177 bariatric surgeries of the SG and RYGB modalities performed from January 2018 to December 2021.

Data were obtained from the management of medical and hospital expenses related to surgical and anesthetic supplies, as well as the amount reimbursed by the funding source to the hospital. It is important to note that patient identification was not accessed; only the financial spreadsheet with the quantity of procedures and the aforementioned data was reviewed, ensuring the confidentiality of personal information.

The RYGB was assessed using the SUS table code for “bariatric surgery by laparoscopy” (04.07.01.038-6). The SG was assessed using the SUS table code for “sleeve gastrectomy” (04.07.01.036-0).

For data analysis, a spreadsheet was created using Microsoft Excel. The spreadsheet was then imported into Statistical Package for the Social Sciences (SPSS) software, version 18, for analysis. Frequencies and percentages of the variables were calculated, and frequency distributions were determined to detail the costs and reimbursements for each evaluated procedure.

## RESULTS

During the analyzed period, the medical and hospital expenses management recorded a total of 177 minimally invasive bariatric surgeries, including SG and RYGB. The hospital registered 81.35% (n=144) of these procedures under the code for “bariatric surgery by laparoscopy” and 18.65% (n=33) as “sleeve gastrectomy,” due to the 10^th^ Revision of the International Classification of Diseases -- CID-10 E66 (obesity).

Regarding the funds allocated by the federal government from the registration of these procedures, since 2018, the tertiary public health service receives R$ 6,145.00 for the “bariatric surgery by laparoscopy” procedure, which includes RYGB, and R$ 4,095.00 for “sleeve gastrectomy.” These amounts are received per procedure and do not account for hospitalization or preand postoperative care (Table 1).

**Table 1:** Breakdown of Costs for Each Surgical Procedure.

Costs component	RYGB		SG	
	Cost	%	Cost	%
Basic surgical kit	1.006,58	9.32	1.006,58	9.86
Stapler	990,00	9.16	990,00	9.69
Stapler reloads	4.001,25	37.05	4.225,00	41.39
Harmonic Scalpel	2.227,50	20.62	2.227,50	21.82
Trocars	226,27	2.09	226,27	2.21
Fouchet bougie	28,64	0.26	28,64	0.28
General anesthesia	891,69	8.25	891,69	8.73
Peridural anesthesia	499,83	4.62	499,83	4.89
Disposable utilities^*^	251,30	2.32	251,30	2.46
Total	10.123,06	-	10.346,81	-
Federal transfer	6.145,00	-	4.095,00	-
Deficit	3.969,06		6.251,81	

* Surgical dressings; gauze; scrub items. RYGB: Roux-en-Y gastric bypass; SG: sleeve gastrectomy.

The average total cost for RYGB was R$ 10,123.06 and, for SG, it was R$ 10,346.81. Given the reimbursement from the funding source, the federal government, the service faces a financial deficit of R$ 3,969.06 for RYGB and R$ 6,251.81 for SG when the procedure code is not reported as “bariatric surgery by laparoscopy” (Table 1).

Regarding the average hospital cost for surgical supplies, RYGB incurred a total of R$ 9,907.54, while SG incurred a total of R$ 9,315.84. In addition to the basic surgical materials kit and orthopedic, prosthetic, and special materials (OPME), the cost of anesthetic supplies was also accounted for, totaling an average of R$ 891.69 per procedure. Furthermore, when an epidural anesthetic block was performed, there was an additional cost of R$ 499.83 to the hospital expenses (Table 1).

Breaking down the costs of each procedure, it is evident that the largest proportion of expenses is related to special materials. For RYGB, materials categorized as OPME represented 66.84% of the total cost, equivalent to R$ 7,218.75. For SG, they represented 72.9% (R$ 7,442.50). It is worth noting that the cost of stapler cartridges was the main contributor to this amount.

## DISCUSSION

In recent decades, there has been a substantial increase in the global prevalence of overweight and obesity. In 2016, data indicated that the average body mass index (BMI) of the Brazilian population was 26.6 kg/m^2^, placing the average population within the overweight range^5^. This scenario is similarly reflected in other countries. In Australia, a 2018 survey revealed that 67% of adults were overweight and 31% were considered obese^5^. This situation has significant economic implications, corresponding to 2.8% of the global GDP^17^.

Regarding the increase in the number of bariatric surgeries, data from the American Society for Metabolic and Bariatric Surgery (ASMBS) indicate a 62% increase in the number of bariatric surgeries in the United States from 2011 to 2019, with a decrease in 2020 due to the COVID-19 pandemic^3^. However, despite this increase in surgical volume, there remains a notable disparity between the population eligible for surgical procedures and the proportion that has access to these treatments, particularly within public financing and healthcare systems. Financial aspects, including the limitations of available funding sources to cover these surgeries, whether public or private, emerge as one of the main obstacles contributing to this inequality^6,12^.

Countries such as Canada, the United States, and the United Kingdom witness less than 1% of eligible patients undergoing bariatric procedures, with an average wait time of five years for Canadians from the start of follow-up. The Brazilian Ministry of Health, in data published in 2021, reports that only 0.3% of patients with an indication for the procedure undergo it within the SUS, and less than 0.01% of these interventions are performed laparoscopically^10,12^.

The analysis of total costs by surgical modality reveals that the average cost of RYGB is R$ 10,799.23, while the average cost of SG is R$ 10,207.53. Research conducted in the United States shows average costs of $12,543 for RYGB and $10,531 for SG, although these figures are from a private healthcare system and include the cost of accommodations and healthcare professionals^6^.

The evaluation of perioperative costs in this study was restricted by several variables, including the completion of part of the preoperative care at external services, the lack of cost estimates for accommodations based on the number of patients, and the payment to healthcare professionals that does not account for service productivity^15^. Estimates based on data from the United States indicate costs of $14,942 for SG compared to $15,016 for RYGB, with no statistically significant difference (p=0.80)^11^.

Regarding specific perioperative costs, it was found that the largest component consists of expenses related to the operating room, representing 41.7% of the total, followed by costs for accommodations and medications, which account for 21.9%, and payments to healthcare professionals, totaling 4.9%^17^.

The financial insufficiency related to funding by the Ministry of Health, as evidenced in this study, is also corroborated by a systematic review encompassing 13 studies conducted in various countries. In the United Kingdom, for example, the national health system sets basic reimbursement rates of $5,771 for SG and $6,602 for RYGB, while the estimated average cost of perioperative care reaches $14,389^2^.

The average total cost for RYGB was R$ 10,123.06, and, for SG, it was R$ 10,346.81. Given the reimbursement from the funding source, the federal government, the service faces a financial deficit of R$ 3,969.06 for RYGB and R$ 6,251.81 for SG when the procedure code is not reported as “bariatric surgery by laparoscopy.”

When comparing the costs for each procedure with the reimbursement amounts from the federal government, there is a deficit of R$ 3,969.06 for RYGB (code “bariatric surgery by laparoscopy”) and R$ 6,251.81 for SG (code “sleeve gastrectomy”). Due to this discrepancy in reimbursement, it was established in 2020 that all obesity surgeries must be billed as “bariatric surgery by laparoscopy,” which has made it difficult to accurately delineate the specific surgical modalities performed based on financial sector data.

Despite the considerable costs associated with bariatric surgery, the intervention represents an improvement in the quality of life for patients who undergo it, as well as resulting in savings in overall healthcare costs^14^. It is estimated that bariatric surgery provides savings ranging from $1,209 to $2,016 per patient due to the reduction in adverse health events and the decreased need for medications to treat comorbidities^13^. Bariatric surgery is responsible for a 78% reduction in the prevalence of hypertension and a 92% reduction in the prevalence of type 2 diabetes mellitus. Additionally, it is associated with increased life expectancy and a higher number of years lived without chronic comorbidities^5,16^. The primary savings result from reduced spending on medications and healthcare professionals to treat diabetes mellitus, followed by hypertension and sleep apnea. A study conducted in New Zealand with 114 patients demonstrated a reduction in medication costs from $1,044 to $274.60 per individual one year after the surgery^4,6^.

When identifying the factors contributing to the operational costs of bariatric surgery, the category of special materials stood out as the one that contributes the most to these expenses. In this context, the cost associated with the use of surgical staplers is particularly notable, emerging as the most burdensome component. A multicenter systematic analysis revealed deficiencies in the detailed evaluation of operational costs, particularly due to the inadequate recording of the materials actually used, a situation that can be extrapolated to the present study^2^. Over a three-year follow-up period involving patients who underwent bariatric surgeries at the University of Wisconsin, it was found that the use of surgical staplers accounted for the largest share of the average total perioperative costs, corresponding to 27.7 and 29.2% of expenses for SG and RYGB surgeries, respectively^11^. Despite the trend of surgical staplers being the primary cost component, the proportion identified represented approximately three times the international average, largely due to the importation of these products into Brazil and the devaluation of the local currency against the U.S. dollar^11^.

This study presents a strictly descriptive analysis aimed at illustrating both the economic impact and the institutional barriers inherent in maintaining a minimally invasive bariatric surgery program within the public health system. Despite the typical limitations of observational and descriptive research, it is crucial to highlight that this analysis addresses a topic frequently neglected in the literature, yet it is one of the primary factors contributing to the disparity between the number of individuals eligible for bariatric surgery and those who actually undergo the intervention.

## CONCLUSIONS

The growing number of patients eligible for surgical treatment of obesity and the consequent increase in the volume of these procedures funded by the SUS result in hospital expenses exceeding the reimbursements from the funding source in federal public hospitals, leading to a negative economic balance.
